# Nutritional Intervention Contributes to the Improvement of Symptoms Related to Quality of Life in Breast Cancer Patients Undergoing Neoadjuvant Chemotherapy: A Randomized Clinical Trial

**DOI:** 10.3390/nu13020589

**Published:** 2021-02-10

**Authors:** Ana Priscilla Silva de Souza, Luciana Câmara da Silva, Ana Paula Trussardi Fayh

**Affiliations:** 1 Postgraduate Program in Nutrition, Health Sciences Center, Federal University of Rio Grande do Norte, Natal 59078-970, Rio Grande do Norte, Brazil; ana.pri_sds@yahoo.com.br; 2 Liga Norteriograndense Contra o Câncer, Natal 59075-740, Rio Grande do Norte, Brazil; lucianaliga@hotmail.com; 3 Postgraduate Program in Health Sciences, Health Sciences Center, Federal University of Rio Grande do Norte, Natal 59078-970, Rio Grande do Norte, Brazil; 4 Departamento de Nutrição, Universidade Federal do Rio Grande do Norte, Avenida Senador Salgado Filho, 3000, Bairro Lagoa Nova, Natal 59078-970, Rio Grande do Norte, Brazil

**Keywords:** neoplasm, diet, quality of life, toxicity, breast cancer

## Abstract

During breast cancer treatment, worsening quality of life (QoL) and the presence of toxicities are common, but healthy eating practices are associated with better clinical results. Thus, this study aims to evaluate the effect of a nutritional intervention on QoL and on gastrointestinal and hematological toxicities resulting from chemotherapy in women with breast cancer. A randomized clinical trial was performed at the beginning of neoadjuvant chemotherapy treatment for women with breast cancer. All participants received nutritional advice on healthy eating practices, but only the intervention group (IG) received an individualized diet plan. The study enrolled 34 women, 19 in the IG and 15 in the control group (CG). During the study, the CG significantly presented a reduction (from 21.6 ± 5.9 kg to 18.8 ± 4.0 kg, *p* = 0.009) in handgrip strength (HGS), while the IG did not present changes in this variable. Regarding QoL, the IG preserved the role function during treatment and presented better results for nausea/vomiting and loss of appetite compared to the CG. In gastrointestinal and hematological toxicities, the IG had lower frequencies of leukopenia and abdominal pain. The nutritional intervention preserved the role function of QoL and HGS, reduced the occurrence of nausea/vomiting, loss of appetite and the frequency of leukopenia and abdominal pain.

## 1. Introduction

Breast cancer is considered one of the most prevalent types of cancer in the female population on a global level, both in developed and developing countries with increasing estimates every year [1]. Among the main risk factors, there are environmental and behavioral factors, reproductive and hormonal history, genetics and heredity [2]. Breast cancer is the most common cause of cancer death for women worldwide, about 70% of these occur in low- and middle-income countries, although it is considered a disease with high cure rates when there are both early detection and treatment strategies [1].

Among the treatment modalities to breast cancer, neoadjuvant chemotherapy, the modality of chemotherapy that occurs before surgical procedures, is performed with the objective of improving conditions for tumor resection and prognostic results, decreasing the risk of local recurrence [3]. However, this systemic therapeutic modality can impair nutritional status [4,5], by reducing food intake due to the higher occurrence of gastrointestinal toxicities including nausea and vomiting [6,7,8]. When these toxicities occur over a prolonged time and in a greater degree of severity, they may cause the interruption of the treatment [9]. Moreover, there is a worsening of the quality of life [10,11,12] which seems to be associated with a less favorable clinical prognosis and increased risk of recurrence [8].

During cancer treatment, monitoring quality of life (QoL) is considered a priority, especially since the diagnosis and treatment cause significant functional and emotional repercussions [13]. When it is associated with undesirable adverse effects, such as chemotherapy- or radiation-related toxicities, this impact on QoL becomes even more significant [14,15]. These toxicities, especially related to the gastrointestinal tract, can be negatively associated with nutritional status, dietary intake and nutrient absorption [4,5,7].

Given this scenario, it is important to adopt lifestyle changes including guidance for a balanced diet according to individual needs, in order to prevent nutritional deficiencies, maintain and/or recover nutritional status and improve QoL, contributing to a better long-term prognosis and, consequently, reducing the frequency of toxicities. Thus, the objective of this study was to evaluate the effect of a dietary intervention on the QoL and on gastrointestinal and hematological toxicities of women with breast cancer, at the beginning of the neoadjuvant chemotherapy treatment. The QoL was selected as the primary outcome. The secondary outcomes included gastrointestinal and hematological toxicities related to chemotherapy and nutritional status. We hypothesized that dietary intervention may contribute to the maintenance or improvement of the QoL during chemotherapy, attenuating the symptoms of toxicity.

## 2. Materials and Methods

### 2.1. Study Design and Population

This is a randomized, parallel-group clinical trial, including adult women newly diagnosed with breast cancer indicated for neoadjuvant chemotherapy at a reference center for cancer treatment in the city of Natal, RN, Brazil. The exclusion criteria for participation in the study were: having undergone some type of cancer treatment (chemotherapy, radiotherapy and/or surgery); presenting concomitant consumptive diseases (AIDS, non-oncological liver diseases, tuberculosis), physical and/or intellectual disability to understand the research stages or to consent their participation; being diagnosed with nutritional risk or undergoing outpatient nutritional follow-up; having the treatment protocol changed, in the place where the therapy was performed; and/or the ones difficult to contact.

The calculation of the sample size was determined based on a previous study [16], in which the global health score of the QoL Questionnaire Core 30 (EORTC QLQ-C30) showed a mean difference of 16.0 in the first cycle of chemotherapy between groups (control vs. intervention with diet). A sample size of 34 subjects (17 in each group) was necessary to provide 80% power with the alpha level of 0.05 (G*Power^®^, version 3.1.9.2; Institute for Experimental Psychology in Dusseldorf, Germany) [17].

The data were collected between the months of November 2018 and December 2019. The study was approved by the Research Ethics Committee from the executing institution (93660618.1.0000.5292), with the consent of the co-participant institution (93660618.1.3001.5293). The protocol for this study is available on a Brazilian clinical trial platform (code RBR-3SHHXS).

### 2.2. Data Collection

The neoadjuvant chemotherapy included the following drugs: doxorubicin, cyclophosphamide and an intravenous antiemetic pattern with ondansetron and dexamethasone on the day of each treatment cycle, prior to the infusion of the chemotherapeutic agents. Each chemotherapy cycle occurred every 21 days, and the number of cycles varied according to the clinical indication of each patient. For standardization purposes, all patients were followed up from baseline (T_0_) before the beginning of the first cycle, during the second (T_1_) and third (T_2_) cycles, until the end of the third one (T_3_).

After accepting to participate in the study, data regarding personal, clinical and sociodemographic characteristics; nutritional, anthropometric and muscle strength assessments; QoL (primary outcome) and gastrointestinal and hematological toxicities (secondary outcomes) were collected at different times.

**Figure 1:**
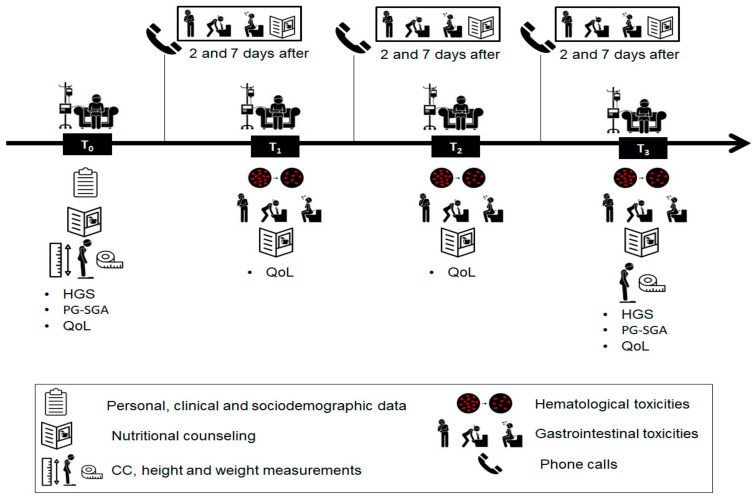
Steps of data collection. Abbreviations: CC, Calf Circumference; HGS, Handgrip strength; QoL, Quality of life; PG-SGA, Patient-Generated Subjective Global Assessment.

Simple computer-based randomization was performed to determine to which treatment group (intervention or control) each participant would be assigned. During T_0_ and T_3_, nutritional and anthropometric data were collected. The quality-of-life data were collected during T_1_, T_2_ and T_3_. Hematological toxicities were collected from the results of laboratory tests performed in routine treatments, during T_1_, T_2_ and T_3_. Gastrointestinal toxicities were collected by telephone calls on the second and seventh day after each treatment cycle, in the intervals between T_0_ and T_1_; T_1_ and T_2_; T_2_ and T_3_ and in person, 21 days after each cycle upon return for the next one, at the moments T_1_, T_2_ and T_3_. The initial nutritional and dietary orientations were conducted at T_0_, possible adjustments in the diet plan of the IG and additional guidance was made at T_1_, T_2_ and T_3,_ and by phone in the calls to register gastrointestinal toxicities. Figure 1 illustrates the steps of data collection.

### 2.3. Nutritional Intervention

At the beginning of the study, two pamphlets designed for this study were given to patients from both groups, which contained (1) nutritional guidelines on healthy eating benefits (e.g., it emphasizes fruits, vegetables, whole grains, and fat-free or low-fat milk products; it includes the intake of lean meats, poultry, fish, beans, and low consumption of food with saturated fats, *trans* fats, cholesterol, salt (sodium), and added sugars); and (2) nutritional information to reduce the severity of chemotherapy-induced nausea and vomiting (e.g., consuming cold foods and eating small meals frequently, avoiding spicy, very sweet, greasy, or fried foods, consuming liquids with ice chips or frozen juice chips, among others). These guidelines were prepared according to Brazilian recommendations for reducing nausea and vomiting through diet [18], and reinforced when necessary at T_1,_ T_2_ and T_3,_ or during phone calls between chemotherapy cycles. Patients in the CG were advised to follow a usual diet based on their food patterns but adopting recommendations from the pamphlets. Only the patients in the IG received a personalized diet with the meal plan estimated individually by a dietitian and based on age, current weight, and height of each subject, recommending between 25 and 30 kcal/kg/day of energy and 1.5 g/kg/day of protein, according to current guidelines [19]. All the other macronutrients and specific micronutrients relevant for women with cancer and overweight (as iron, sodium, calcium, and antioxidants vitamins A, C and E) were offered in order to reach 100% of the recommendations proposed by the Dietary Reference Intakes (DRIs) [20], and no patient required micronutrient dietary supplementation to achieve an intake goal. At the beginning of each new cycle of chemotherapy, an in-person orientation on nutrition was performed for all participants in order to reinforce these instructions. Supplementary Table S1 shows data on food consumption from both groups through the period.

### 2.4. Nutritional Assessment

The nutritional assessment included the application of the Patient-Generated Subjective Global Assessment (PG-SGA) [21] and of the anthropometric and muscle strength assessment. Body mass (kg) and height (cm) were measured for the calculation of the Body Mass Index (BMI) and its classification was performed according to the World Health Organization [22]. Calf circumference (CC) (cm) was also measured and low CC was considered when >33 cm [23]. Muscle strength was obtained through handgrip strength (HGS). We considered the highest value among three measurements performed with a Jamar^®^ mechanical dynamometer (Seoul, Korea), with an accuracy of 0.5 kg. The reference value was considered according to the European Working Group on Sarcopenia in Older People (EWGSOP), with the cut-off point for dynapenia < 16 kgF in women [24].

### 2.5. Quality of Life Assessment

The QoL assessment was conducted based on the application of the European Organization for Research and Treatment of Cancer QLQ C-30 [25]. The conversion of the responses obtained into scores for each domain was conducted according to guidelines from the EORTC QLQ-C30 Scoring Manual, 3rd edition [26].

### 2.6. Toxicity Assessment

The evaluation of gastrointestinal toxicities (nausea, vomiting, diarrhea, anorexia, abdominal pain, constipation, mucositis and weight loss) was obtained through self-reporting during the follow-up (Figure 1) and hematological toxicities (anemia, leucopenia, neutropenia and thrombocytopenia) were obtained by evaluating the results of laboratory tests performed in routine treatments. Both were graded according to the Common Terminology Criteria for Adverse Events (CTCAE) | Protocol—National Cancer Institute version 5.0 [27]. This document classifies toxicities in five grades, according to severity (e.g., 0 represents absence and 5 is the most serious degree of toxicity). In this study, we considered that toxicity was present in any values above zero. The dose-limiting toxicity (DLT) was defined as the need to reduce the drug dose, to delay or definitively discontinue the protocol.

### 2.7. Statistical Analysis

Data analysis was performed using the IBM SPSS Statistics, version 25 (IBM^®^, Chicago, IL, USA). Data normality was tested using the Shapiro–Wilk test. The categorical data are presented in absolute and relative frequency. The chi-squared test was used to compare the proportion between the intervention and the control group. Fisher’s exact test was used when the assumption of the expected count was violated. The Z-test was used to identify possible differences in proportions between groups. The results were presented as mean ± standard deviation for parametric data and median (25th and 75th percentiles) for nonparametric data. An intention-to-treat analysis was adopted. The paired-samples *t*-test was used to compare BMI, HGS and CC in each group at the beginning and end of the study. The generalized estimation equation (GEE) was used to verify the effect of group interaction (two factors: intervention vs. control) and time (four factors: pre-chemotherapy and three cycles) for the outcome QoL. The GEE model for each variable was defined by the fit quality of the model. The normality of the residues was verified by the Q-Q normality graph. The GEE Tweedie model was used for the variables that presented zero sample values. Cohen’s d standardized measure (Cohen’s d_s_) was used to verify the effect size of the means, and it was interpreted as small (*d* = 0.2), medium (*d* = 0.5), and large effect size (*d* = 0.8) [28]. The accepted significance level was *p* < 0.05.

## 3. Results

**Figure 2:**
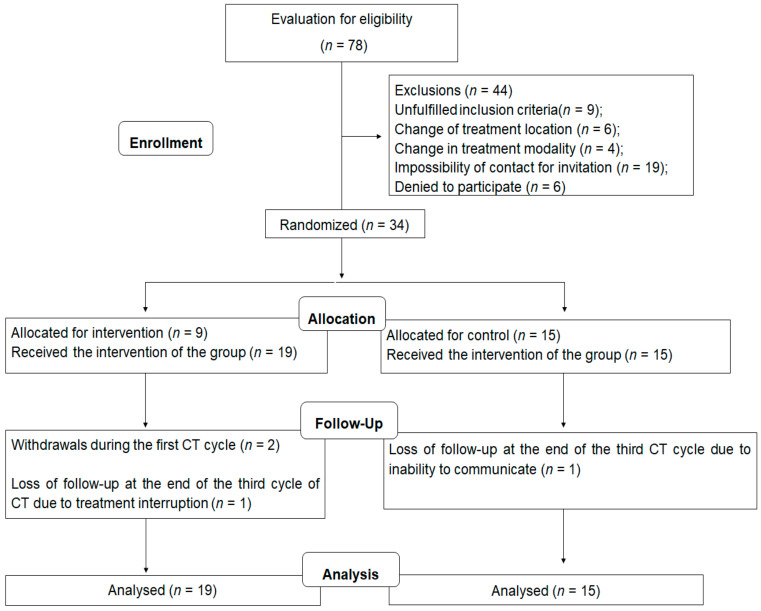
Flowchart of the randomized clinical trial.

The recruitment and follow-up processes are presented in Figure 2. During data collection, 78 patients were evaluated for eligibility and 34 were randomized, with a total of 19 participants in the IG and 15 in the CG. With an intention-to-treat analysis, all patients were analyzed at the end of the period. The median of the time for general follow-up of the participants was of 63 (61–70) days. The mean age at menarche of the patients was of 12.9 ± 1.7 years, and the prevalence of hypertension and diabetes was 8.8%, for each disease. Table 1 shows the sociodemographic and clinical characteristics of the participants at the beginning of the study, with no differences between groups.

Nutritional, anthropometric and HGS measurements of patients at baseline (T_0_) and at the end (T_3_) of the study are shown in Table 2. It was observed that the CG significantly had their HGS reduced, while the IG maintained this measure. No differences were observed in other variables.

**Figure 3:**
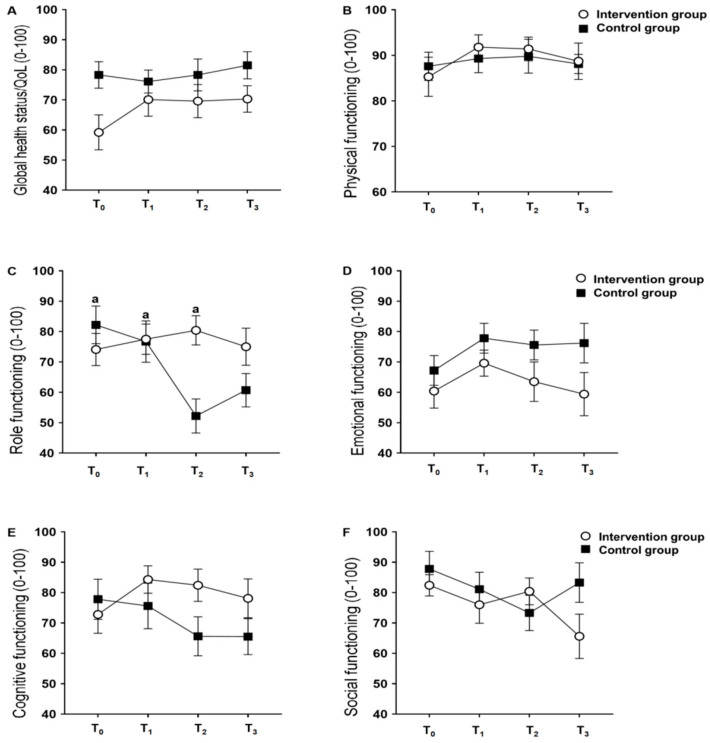
Comparison of global health/QoL and functional scales by the EORTC QLQ-C30 between the nutritional intervention and control groups throughout the study. (**A**) Global health status/QoL; (**B**) Physical functioning; (**C**) Role functioning; (**D**) Emotional functioning; (**E**) Cognitive functioning; (**F**) Social functioning. Abbreviations: Data presented as mean and standard deviation. T_0_, study baseline, pre-chemotherapy moment; T_1_, regarding the end of the first chemotherapy cycle; T_2_, regarding the end of the second chemotherapy cycle; T_3_, regarding the end of the third cycle of chemotherapy. ^a^ represents a statistical difference from T2 in the control group (*p* = 0.004).

Figure 3 shows the results of global health status/QoL and functional domains. The results of global health, physical function, emotional function, cognitive function, and social function did not show major effects of group and time, and group and time interaction, respectively. However, role function showed the effect of interaction between group and time (*p* < 0.001), and the main effect of time (*p* = 0.004), without the main effect of the group (*p* = 0.103). The role function of the CG in the second chemotherapy cycle was lower than in the pre-chemotherapy and in the first cycle for the same group, and lower than in the first and second cycles for the IG (Figure 3C).

The symptom scales by EORTC QLQ-C30 over the chemotherapy cycles are shown in Table 3. The nausea/vomiting scale showed an interaction effect between group and time (*p* < 0.001), and the main effect of time (*p* = 0.018), without the main effect of the group (*p* = 0.065), with a large effect size measured by Cohen’s d_s_ (>0.8 for all times). The scale for loss of appetite also showed statistical significance, but only for the main effect of time (*p* < 0.001), with a large effect size measured by Cohen’s d_s_ (>0.8 for T_0_, T_2_ and T_3_ cycles). A reduction in nausea and vomiting scores was observed in the IG during follow-up, while in the CG an increase was observed. The IG showed reduced scores for loss of appetite over the follow-up period, between the third cycle of chemotherapy and the pre-chemotherapy.

The frequencies of hematological and gastrointestinal toxicities in the IG and CG in the first three cycles of neoadjuvant chemotherapy are shown in Table 4. The IG had a lower frequency of leukopenia in the third cycle of chemotherapy (*p* = 0.034) and a lower frequency of abdominal pain in the second cycle (*p* = 0.034) compared to the CG. There was no difference in other frequency variables analyzed between the groups during the chemotherapy cycles.

## 4. Discussion

Studies that evaluate the effects of dietary intervention in patients with breast cancer undergoing chemotherapy are still scarce, and to our knowledge, this is the first study that evaluates the effect of a dietary intervention on the QoL and on gastrointestinal and hematological toxicities of women with breast cancer starting neoadjuvant chemotherapy. A recent study made similar assessments, however, it was restricted to the observation of nausea and vomiting using a specific scale, and its population was composed of women undergoing adjuvant treatment, which is when chemotherapy treatment occurs after the surgical procedure [16]. Thus, this study contributes to the literature, expanding knowledge about the role of nutrition in the treatment of breast cancer, showing the positive, although modest, effects on nutritional status, QoL and reduction of toxicity during the start of neoadjuvant chemotherapy.

Regarding QoL, it was observed that the role function was better maintained in the IG throughout the chemotherapy sessions (*p* < 0.001 for interaction). Similar results have been observed in previous studies [28,29] in women with breast cancer but with different therapeutic diets. The role function is related to the performance of daily activities and, consequently, to the autonomy of these women, and the preservation of this functional scale represents an important factor for adherence and reduction of dropout rates throughout the treatment of breast cancer [30]. The other global health/QoL results and functional scales demonstrated no differences between groups, which differs from the results found in a recent clinical trial, showing that nutritional intervention during adjuvant treatment improved overall health/QoL scores and physical, emotional, cognitive and role functions [23]. We believe that the fact that both groups were instructed interfered with the non-existence of differences between them for overall health/QoL and functional scales, since it is proven that chemotherapy has a negative impact on the QoL of cancer patients [24,31]. Again, we reinforce that the previous studies mentioned were carried out in women with breast cancer and with different chemotherapy regimens than that of the present study.

The symptom scales, assessed based on the EORTC QLQ-C30 questionnaire, showed significant results only in nausea/vomiting and loss of appetite scores, unlike the study by Najafi et al., who observed worse results in the CG for fatigue, nausea/vomiting, pain, dyspnea, loss of appetite, constipation and diarrhea [16]. However, it is worth mentioning the methodological differences between studies, especially regarding therapeutic modalities, the time that the patients were under treatment and the fact that all patients in this study received some type of nutritional orientation, which in the clinical trial conducted by Najafi et al. was restricted to patients in the IG.

Regarding hematological toxicities related to chemotherapy, the results of the present study showed that the IG had lower frequencies of leukopenia in the third cycle of chemotherapy and of abdominal pain in the second cycle of chemotherapy compared to the CG. Hematological toxicities induced by chemotherapy, such as leukopenia, were not defined as the primary outcome of this study, and probably the small sample size was not sufficient to eliminate type 1 error. In addition, individuals undergoing multiple types of treatment or who have compromised nutritional status, especially in the presence of changes in body composition, such as sarcopenia and cachexia, tend to have higher degrees of toxicity during chemotherapy, Refs. [4,32,33,34,35] which was not observed in the studied population, who, for the most part, were overweight, without significant changes in CC, which is consistent with the literature about the nutritional status of women with breast cancer [32,33]. In addition, the participants in the present study were submitted to a unique therapeutic modality, without having undergone other types of cancer treatments, which may contribute to the long-term impairment of nutritional status.

Furthermore, our findings point to the protective effect of nutritional and dietary intervention in maintaining HGS, as observed in the IG. Although the main outcome of the study was not to evaluate the effects of the intervention on nutritional status and body composition, this finding corroborates the positive results of the nutritional intervention on role function evaluated as an aspect of QoL. A previous study showed that the strength of the upper limbs in women diagnosed with breast cancer is reduced [36] and this highlights the importance of measuring the HGS in this public, which can correlate with the loss of muscle mass [37] as well as being associated with musculoskeletal symptoms [38,39], reduced physical function [38,40], increased occurrence of adverse events [39] and/or complications during treatment [41], therapy change [42] and higher mortality [43,44] for various types of cancer. The relationship between the preservation of muscle strength and QoL is indisputable, demonstrating another benefit of adequate nutrition as a positive agent for maintaining nutritional status and, consequently, physical function.

The presence of a DLT was considered low, especially when compared to other results from our research group [4]. We found a frequency of 23.3%, 20.7% and 22.2% for DLT in the first three cycles of chemotherapy, respectively, in 60 patients with cancer in the gastrointestinal tract [4], while in this study only two patients presented DLT (less than 6% of the total sample). This difference can be attributed to the different site of the tumor (gastrointestinal cancer), to a more deteriorated nutritional status in the previous study, and to the fact that these patients were not necessarily undergoing neoadjuvant chemotherapy, having already undergone other types of treatment.

This study is a pioneer in showing the positive effects of nutritional intervention during the onset of neoadjuvant chemotherapy in patients with breast cancer. Thus, the absence of methodologically similar studies makes it difficult to compare findings. Limitations regarding the small sample size and limited follow-up time should also be considered, since it is a single-center study and the indication for neoadjuvant chemotherapy treatment, in addition to not being the most frequent in this center, presents individual variation after the third cycle, according to the clinical characteristics of the patients. Therefore, complementary studies with a larger number of participants are needed to confirm these preliminary findings. Another limitation refers to the fact that both groups received nutritional counseling, as it would be unethical not to offer any orientation to the control group on how to minimize the adverse effects of chemotherapy. Finally, it should be noted that this study was conducted with women with breast cancer, and the results should not be extrapolated to male individuals with breast cancer and/or female populations with other types of cancer.

However, the present study also has strengths. Although the population was small, it was homogeneous and with no significant clinical differences at the beginning of the study, in addition to not having undergone any other types of previous cancer treatment, which could generate nutritional impairment and consequently interfere with the quality of the results for QoL and toxicities. The intervention was conducted with the prescription of an individualized diet plan, which met nutritional needs, according to the specific recommendations, for cancer patients, associated with nutritional guidelines for the improvement of signs and symptoms arising from the chemotherapy treatment. As most studies that evaluate adverse effects of chemotherapy treatment have a retrospective design, the performance of a clinical trial collaborates with the literature regarding non-pharmacological strategies that can effectively reduce adverse effects of cancer treatments.

## 5. Conclusions

In conclusion, the results of this study indicate that a nutritional intervention during the first three cycles of neoadjuvant chemotherapy in women with breast cancer was able to preserve a QoL domain related to role function, producing positive effects regarding the occurrence of nausea/vomiting and loss of appetite and reducing the frequency of leukopenia and abdominal pain during treatment. These findings reinforce that nutritional interventions during chemotherapy treatments are important to minimize possible nutritional impacts, consequent clinical involutions during therapy or even treatment interruption. In addition, we emphasize the need for complementary studies with patients also undergoing neoadjuvant treatment to better support and guide assistance and nutritional care strategies for this population.

## Figures and Tables

**Table 1:** Sociodemographic, nutritional and clinical characteristics of patients at the beginning of the study.

Variables	Intervention Group (*n* = 19)	Control Group (*n* = 15)	*p*
**Age (years)**	44.3 ± 9.2	45.5 ± 8.6	0.697
**Ethnicity, *n* (%)**			
Caucasian	5 (62.5)	3 (37.5)	1.000
Non-Caucasian	14 (53.8)	12 (46.2)	
**Education, *n* (%)**			
Up to elementary school	4 (50.0)	4 (50.0)	1.000
High school/Higher	15 (57.7)	11 (42.3)	
**Income**			
Up to 1 MW	7 (53.8)	6 (46.2)	0.933
>1 MW	10 (58.8)	7 (41.2)	
Not informed	2 (50.0)	2 (50.0)	
**Reproductive history, *n* (%)**			
With children	14 (48.3)	15 (51.7)	0.053
Without children	5 (100.0)	0 (0.0)	
**Breastfeeding, *n* (%)**			
Yes	13 (52.0)	12 (48.0)	0.697
No	6 (66.7)	3 (33.3)	
**Menopause, *n* (%)**			
Yes	5 (55.6)	4 (44.4)	1.000
No	14 (41.2)	11 (44.0)	
**Physical activity, *n* (%)**			
Perform	3 (60.0)	2 (40.0)	1.000
Do not perform	16 (55.2)	13 (44.8)	
**PG-SGA, *n* (%)**			
A	17 (60.7)	11 (39.4)	0.370
B e C	2 (33.3)	4 (66.7)	
**BMI classification, *n* (%)**			
Low weight	0 (0.0)	1 (100.0)	0.371
Eutrophic	6 (60.0)	4 (40.0)	
Overweight	10 (66.7)	5 (33.3)	
Obesity	3 (37.5)	5 (62.5)	
**Reduced HGS (<16 kgF), *n* (%)**			
Yes	2 (40.0)	4 (60.0)	1.000
No	16 (55.2)	13 (44.8)	
**Reduced CC (<33 cm), *n* (%)**			
Yes	2 (33.3)	4 (66.7)	0.336
No	9 (64.3)	5 (35.7)	
**Comorbidities, *n* (%)**			
With comorbidities	3 (60.0)	2 (40.0)	1.000
No comorbidities	16 (55.2)	13 (44.8)	
**Staging (TNM)**			
II	12 (54.5)	10 (45.5)	0.666
III	6 (54.5)	5 (45.5)	
Undefined	1 (100.0)	0 (0.0)	
**Molecular subtype**			
Luminal A	5 (62.5)	3 (37.5)	0.481
Luminal B	9 (60.0)	6 (40.0)	
HER2+	3 (75.0)	1 (25.0)	
Negative triple	1 (20.0)	4 (80.0)	
Undefined	1 (50.0)	1 (50.0)	

Data presented as mean ± standard deviation, and absolute and relative frequency (%). CC, Calf Circumference; HGS, Handgrip strength; MW, Minimum Wage; PG-SGA, Patient-Generated Subjective Global Assessment.

**Table 2:** Nutritional, anthropometric and handgrip strength measurements of patients at the baseline (T_0_) and at the end (T_3_) of the study.

Variables	Intervention Group (*n* = 19)			Control Group (*n* = 15)		
	T_0_	T_3_	*p*	T_0_	T_3_	*p*
Weight (kg)	63.8 ± 5.7	62.9 ± 5.7	0.189	69.4 ± 19.4	68.4 ± 18.2	0.184
BMI (kg/m^2^)	26.9 ± 2.5	25.8 ± 2.7	0.215	27.8 ± 6.1	27.4 ± 5.8	0.245
CC (cm)	34.6 ± 2.2	34.4 ± 2.1	0.319	36.1 ± 5.7	35.9 ± 5.4	0.89
HGS (kgF)	19.2 ± 4.6	17.8 ± 4.9	0.125	21.6 ± 59	18.8 ± 4.0	0.009

Data presented as mean ± standard deviation. BMI, Body Mass Index; CC, Calf Circumference; HGS, Handgrip strength.

**Table 3:** Comparison of symptom scales by the EORTC QLQ-C30 between the nutritional intervention group and the control group in the pre-chemotherapy period and in the first three cycles of neoadjuvant chemotherapy.

Symptom Scales	Intervention Group (*n* = 19)				Control Group (*n* = 15)				*p*_Interaction_	*p*_time_	*p*_group_
	T_0_	T_1_	T_2_	T_3_	T_0_	T_1_	T_2_	T_3_			
Fatigue	27.0 ± 3.8	28.6 ± 7.0	27.2 ± 6.1	32.1 ± 7.3	29.3 ± 7.5	26.7 ± 5.5	26.9 ± 4.2	33.3 ± 5.2	0.948	0.446	0.962
Cohen’s d_s_	0.4	0.3	0.06	0.19							
Nausea/vomiting	28.6 ± 5.5	44.4 ± 22.7	33.3 ± 0.1	16.7 ± 0.1	27.8 ± 9.1	16.7 ± 0.1 ^a^	21.4 ± 2.8 ^a^	23.3 ± 3.7	**<0.001**	**0.018**	0.065
Cohen’s d_s_	0.11	**1.64**	**6.37**	**2.67**							
Pain	38.1 ± 6.2	28.6 ± 8.7	41.7 ± 9.3	43.7 ± 9.3	38.9 ± 9.4	26.7 ± 7.9	45.8 ± 16.0	38.1 ± 8.0	0.936	0.653	0.932
Cohen’s d_s_	0.10	0.23	0.32	0.64							
Insomnia	63.3 ± 7.4	53.3 ± 7.3	53.3 ± 11.9	52.4 ± 9.2	73.3 ± 11.2	61.9 ± 10.5	70.8 ± 7.1	52.4 ± 6.2	0.872	0.266	0.160
Cohen’s d_s_	**1.08**	0.97	**1.74**	0.00							
Financial difficulties	83.3 ± 5.6	83.3 ± 7.1	81.0 ± 6.5	77.8 ± 7.2	63.0 ± 9.7	70.4 ± 9.7	74.1 ± 10.2	59.3 ± 10.2	0.498	0.275	0.108
Cohen’s d_s_	**2.64**	**2.43**	**0.83**	**2.14**							
Appetite loss	80.0 ± 11.9	33.3 ± 0.1 ^b^	50.0 ± 11.8	33.3 ± 0.1 ^b^	50.0 ± 10.4	33.3 ± 0.1	41.7 ± 7.2	38.9 ± 5.1	0.199	**<0.001**	0.173
Cohen’s d_s_	**2.66**	0.00	**0.83**	**1.65**							
Dyspnea	0.0 (0.0 a 0.0)	0.0 (0.0 a 0.0)	0.0 (0.0 a 0.0)	0.0 (0.0 a 0.0)	0.0 (0.0 a 0.0)	0.0 (0.0 a 0.0)	0.0 (0.0 a 16.7)	0.0 (0.0 a 0.0)	-	-	-
Constipation	0.0 (0.0 a 0.0)	0.0 (0.0 a 0.0)	0.0 (0.0 a 0.0)	0.0 (0.0 a 0.0)	0.0 (0.0 a 33.3)	0.0 (0.0 a 33.3)	0.0 (0.0 a 0.0)	0.0 (0.0 a 0.0)	-	-	-
Diarrhea	0.0 (0.0 a 33.3)	0.0 (0.0 a 0.0)	0.0 (0.0 a 0.0)	0.0 (0.0 a 0.0)	0.0 (0.0 a 0.0)	0.0 (0.0 a 0.0)	0.0 (0.0 a 0.0)	0.0 (0.0 a 0.0)	-	-	-

Data presented as mean and standard deviation and median. T_0_, study baseline, pre-chemotherapy moment; T_1_, regarding the end of the first chemotherapy cycle; T_2_, regarding the end of the second chemotherapy cycle; T_3_, regarding the end of the third cycle of chemotherapy. ^a^, statistically different from T_2_ in the intervention group. ^b^, statistically different from T_0_ of the intervention group. Cohen’s ds, effect size of the means of comparison between groups in the same cycle (times). In bold: statistically significant differences.

**Table 4:** Comparison of the frequency of hematological and gastrointestinal toxicities and the presence of dose-limiting toxicity between the intervention and control groups in the first three cycles of neoadjuvant chemotherapy.

Variables		T_1_			T_2_			T_3_		
		IG (*n* = 19)	CG (*n* = 15)	*p*	IG (*n* = 19)	CG (*n* = 15)	*p*	IG (*n* = 19)	CG (*n* = 15)	*p*
**Hematological toxicities**										
**Anemia**										
Yes		5 (41.7)	7 (58.3)	0.218	8 (47.1)	9 (52.9)	0.464	11 (52.4)	10 (47.6)	0.907
No		14 (63.6)	8 (36.4)		9 (60.0)	6 (40.0)		6 (54.5)	5 (45.5)	
**Leukopenia**										
Yes		7 (58.3)	5 (41.7)	0.832	5 (62.5)	3 (37.5)	0.691	3 (27.3)	8 (72.7)	**0.034**
No		12 (54.5)	10 (45.5)		12 (50.0)	12 (50.0)		14 (66.7)	7 (33.3)	
**Neutropenia**										
Yes		7 (70.0)	3 (30.0)	0.451	4 (80.0)	1 (20.0)	0.338	2 (28.6)	5 (71.4)	0.209
No		12 (50.0)	12 (50.0)		13 (48.1)	14 (51.9)		15 (60.0)	10 (40.0)	
**Thrombocytopenia**										
Yes		0 (0.0)	1 (100.0)	0.441	-	-	-	0 (0.0)	1 (100.0)	0.469
No		19 (57.6)	14 (42.4)		15 (46.9)	17 (53.1)		17 (54.8)	14 (45.2)	
**Gastrointestinal toxicities**										
**Nausea**										
Yes		19 (55.9)	15 (44.1)	-	16 (51.6)	15 (48.4)	1.000	16 (51.6)	15 (48.4)	1.000
No		-	-		1 (100.0)	0 (0.0)		1 (100.0)	0 (0.0)	
**Vomiting**										
Yes		10 (45.5)	12 (54.5)	0.097	3 (50.0)	3 (50.0)	1000	3 (37.5)	5 (62.5)	0.423
No		9 (75.0)	3 (25.0)		14 (53.8)	12 (46.2)		14 (58.3)	10 (41.7)	
**Diarrhea**										
Yes		2 (33.3)	4 (66.7)	0.370	1 (50.0)	1 (50.0)	1.000	-	-	-
No		17 (60.7)	11 (39.3)		16 (53.3)	14 (46.7)		17 (53.1)	15 (46.9)	
**Anorexia**										
Yes		11 (52.4)	10 (47.6)	0.728	11 (52.4)	10 (47.6)	0.907	8 (44.4)	10 (55.6)	0.265
No		8 (61.5)	5 (38.5)		6 (54.5)	5 (45.5)		9 (64.3)	5 (35.7)	
**Abdominal pain**										
Yes		7 (43.8)	9 (56.3)	0.179	3 (27.3)	8 (72.2)	**0.034**	2 (28.6)	5 (71.4)	0.209
No		12 (66.7)	6 (33.3)		14 (66.7)	7 (33.3)		15 (60.0)	10 (40.0)	
**Constipation**										
Yes		5 (38.5)	8 (61.5)	0.107	7 (50.0)	7 (50.0)	0.755	2 (25.0)	6 (75.0)	0.106
No		14 (66.7)	7 (33.3)		10 (55.6)	8 (44.4)		15 (62.5)	9 (37.5)	
**Mucositis**										
Yes		0 (0.0)	2 (100.0)	0.187	3 (42.9)	4 (57.1)	0.678	3 (42.9)	4 (57.1)	0.678
No		19 (59.4)	13 (40.6)		14 (56.0)	11 (44.0)		14 (56.0)	11 (44.0)	
**Weight loss**										
Yes		1 (100.0)	0 (0.0)	1.000	-	-	-	0 (0.0)	1 (100.0)	0.469
No		18 (54.5)	15 (45.5)		17 (53.1)	15 (46.9)		17 (54.8)	14 (45.2)	
**Presence of dose-limiting toxicity, *n* (%)**										
Yes		0 (0.0)	2 (100.0)	0.187	3 (100.0)	0 (0.0)	0.229	0 (0.0)	2 (100.0)	0.212
No		19 (59.4)	13 (40.6)		14 (48.3)	15 (51.7)		17 (56.7)	13 (43.3)	

Data presented in absolute and relative frequency (%). CG, Control Group; IG, Intervention Group; T_1_, regarding the end of the first chemotherapy cycle; T_2_, regarding the end of the second chemotherapy cycle; T_3_, regarding the end of the third cycle of chemotherapy. In bold: statistically significant differences.

## Data Availability

Data available on request due to restrictions e.g., privacy or ethical. The data presented in this study are available on request from the corresponding author. The data provided by the volunteers are not publicly available due to privacy/ethical restrictions.

## Supplementary Materials

The following are available online at https://www.mdpi.com/2072-6643/13/2/589/s1, Table S1: Comparison of dietary intake between nutritional intervention and control groups in the first three cycles of neoadjuvant chemotherapy.
