# Supplementary material for: Nutritional Intervention Contributes to the Improvement of Symptoms Related to Quality of Life in Breast Cancer Patients Undergoing Neoadjuvant Chemotherapy: A Randomized Clinical Trial
Source: Nutrients. 2021 Feb 10;13(2):589. doi: 10.3390/nu13020589 (PMC7916763; doi:10.3390/nu13020589)
Supplement: Supplementary file 1 [file nutrients-13-00589-s001.pdf]

**Table S1.** Comparison of dietary intake between nutritional intervention and control groups in the first three cycles of neoadjuvant chemotherapy.

| Variables | Intervention Group ($n$ = 19) | | | Control Group ($n$ = 15) | | | $p_{Interaction}$ | $p_{time}$ | $p_{group}$ |
|---|---|---|---|---|---|---|---|---|---|
| | $T_1$ | $T_2$ | $T_3$ | $T_1$ | $T_2$ | $T_3$ | | | |
| Energy (kcal/day) | 1603.1 ± 107.9 | 1575.5 ± 103.4 | 1672.9 ± 96.9 | 1710.0 ± 112.5 | 1710.7 ± 163.8 | 1742.1 ± 240.1 | 0.917 | 0.761 | 0.526 |
| Carbohydrate (g/day) | 221.3 ± 13.5 | 217.0 ± 13.7 | 241.2 ± 22.6 | 219.5 ± 19.3 | 231.2 ± 19.1 | 224.7 ± 37.1 | 0.512 | 0.751 | 0.965 |
| Protein (g/day) | 79.2 ± 8.8 | 71.7 ± 7.0 | 83.7 ± 9.7 | 90.9 ± 8.8 | 84.1 ± 11.8 | 92.0 ± 12.1 | 0.951 | 0.457 | 0.245 |
| Fat (g/day) | 45.0 ± 5.9 | 42.6 ± 4.2 | 42.7 ± 3.8 | 52.2 ± 5.2 | 50.5 ± 7.1 | 51.4 ± 7.4 | 0.986 | 0.934 | 0.129 |
| Cholesterol (mg/day) | 357.3 ± 50.2 | 325.3 ± 57.5 | 314.5 ± 35.5 | 373.9 ± 52.9 | 315.3 ± 47.9 | 484.6 ± 71.2 | 0.262 | 0.382 | 0.244 |
| Saturated fat (g/day) | 16.3 ± 2.9 | 15.0 ± 1.4 | 13.9 ± 1.6 | 17.2 ± 2.1 | 16.0 ± 2.4 | 17.5 ± 3.6 | 0.701 | 0.862 | 0.422 |
| Monounsaturated fat (g/day) | 14.4 ± 2.3 | 13.0 ± 1.8 | 12.4 ± 1.4 | 17.2 ± 2.2 | 15.9 ± 2.6 | 15.8 ± 2.1 | 0.967 | 0.639 | 0.099 |
| Polyunsaturated fat (g/day) | 7.3 ± 0.8 | 7.0 ± 0.8 | 8.2 ± 1.2 | 8.8 ± 1.0 | 10.8 ± 1.6 | 10.0 ± 1.7 | 0.506 | 0.653 | 0.376 |
| Fiber (g/day) | 23.4 ± 2.4 | 21.5 ± 1.8 | 21.9 ± 2.5 | 20.3 ± 2.8 | 23.7 ± 3.6 | 20.6 ± 2.8 | 0.407 | 0.781 | 0.804 |
| Sodium (mg/day) | 1557.6 ± 209.0 | 1709.5 ± 216.3 | 1587.6 ± 176.6 | 2085.4 ± 420.9 | 1873.2 ± 268.5 | 2138.4 ± 481.0 | 0.614 | 0.977 | 0.140 |
| Potassium (mg/day) | 3026.0 ± 325.1 | 2920.3 ± 243.0 | 2997.7 ± 355.6 | 2743.1 ± 338.8 | 3097.3 ± 366.5 | 2684.5 ± 323.1 | 0.403 | 0.690 | 0.714 |
| Zinc (mg/day) | 8.9 ± 1.0 | 8.1 ± 1.2 | 8.1 ± 0.9 | 9.8 ± 1.3 | 9.6 ± 1.3 | 8.8 ± 1.3 | 0.903 | 0.711 | 0.360 |
| Selenium (mg/day) | 68.3 ± 10.5 | 73.4 ± 10.7 | 79.9 ± 21.3 | 76.6 ± 13.9 | 65.9 ± 12.0 | 98.4 ± 14.8 | 0.698 | 0.461 | 0.633 |
| Piridoxina (mg/day) | 1.6 ± 0.2 | 1.5 ± 0.2 | 1.2 ± 0.1 | 1.4 ± 0.2 | 1.6 ± 0.2 | 1.6 ± 0.3 | 0.272 | 0.648 | 0.761 |

Data presented in mean ± standard deviation. T1, regarding the end of the first chemotherapy cycle; T2, regarding the end of the second chemotherapy cycle; T3, regarding the end of the third cycle of chemotherapy.
